# Weak Hearts

**Published:** 1889-09

**Authors:** 


### WEAK HEARTS.

A weak heart seems to be decidedly more practically inconvenient than a weak head. If a man or a woman be a little feeble about the region of the brain, it is generally of little moment. Some post or other will be provided if the conduct be respectable; and lack of brains is too common to excite any particular attention either in the person concerned or in those about him. But a weak heart insists upon putting itself in evidence at all sorts of convenient and inconvenient times. If its possessor finds himself rather late for his morning train, and makes a "spurt" to recover lost time, the exertion is usually followed by such a "bad quarter of an hour" that he resolves in future rather to lose a dozen trains than to risk temporary suffocation or permanent syncope again. The practical evils which are associated with a feeble heart are innumerable, and will readily suggest themselves to those who possess so unsatisfactory a pumping engine. Weak hearts are by no means so common as is often supposed. Many a man who thinks he has got one is merely dyspeptic; many a woman owes her symptoms to tight lacing or insufficient feeding. If the dyspeptic be cured, or the tight lacing be dispensed with, the symptoms of heart weakness will disappear. Even when the heart is genuinely "weak" the weakness is not always due to special disease of that organ. It may be only part of a general weakness of the whole system, which is easily curable.

